# Evaluating the Safety of Creatine Monohydrate in Adolescents: A Systematic Review of Renal, Hepatic, and Cardiometabolic Outcomes

**DOI:** 10.7759/cureus.106808

**Published:** 2026-04-10

**Authors:** Allen Rubinchuk, Zachariah Dulkoski, Nadiya A Persaud, Michelle Wallen

**Affiliations:** 1 Research, Orlando College of Osteopathic Medicine, Winter Garden, USA; 2 College of Public Health, University of South Florida, Tampa, USA; 3 Emergency Medicine, UCF Lake Nona Hospital, Orlando, USA

**Keywords:** adolescent athletes, creatine monohydrate, dietary supplementation, renal safety, sports nutrition

## Abstract

Creatine monohydrate is a widely used dietary supplement for performance enhancement among athletes and physically active individuals. Questions regarding its safety in adolescent populations remain an important consideration for clinicians and families. This systematic review evaluates the safety of creatine monohydrate supplementation in adolescent athletes and physically active youth. A comprehensive search of PubMed was conducted in accordance with Preferred Reporting Items for Systematic Reviews and Meta-Analyses (PRISMA) guidelines to identify relevant human studies published between 2015 and 2025. Inclusion criteria required original research involving adolescents or physically active youth receiving creatine monohydrate with reported safety outcomes. Five studies met eligibility criteria and were included in the final analysis, comprising randomized controlled trials and longitudinal cohort studies. Across diverse populations, including youth athletes and adolescents with medical conditions, creatine supplementation was generally well tolerated, with no consistent short-term safety signals reported in renal function, liver enzymes, or cardiometabolic risk markers within the study periods. No serious adverse events were attributed to supplementation. These findings summarize currently available evidence regarding creatine safety in adolescent and physically active pediatric populations and highlight the need for larger prospective studies with standardized dosing protocols and longer follow-up periods.

## Introduction and background

Creatine monohydrate is a naturally occurring nitrogen-containing compound synthesized endogenously from arginine, glycine, and methionine and stored primarily in skeletal muscle as phosphocreatine, where it plays a critical role in rapid adenosine triphosphate (ATP) regeneration during high-intensity, short-duration exercise [1,2]. Dietary creatine intake is derived predominantly from animal-based foods, and supplementation with creatine monohydrate has been extensively studied in adult athletic populations. In these populations, creatine supplementation consistently demonstrates improvements in muscular strength, power output, and lean body mass, alongside a well-established safety profile across both short- and long-term use [3-5].

Creatine monohydrate is among the most commonly used dietary supplements in adolescent and young adult athletes, with reported prevalence estimates ranging from approximately 5% to more than 30%, depending on age, sex, sport, and competitive level [6,7]. Despite its widespread use, creatine supplementation in individuals younger than 18 years remains controversial. Ongoing concerns include potential effects on renal and hepatic function, cardiovascular risk, and the relative paucity of long-term randomized safety data in pediatric and adolescent populations [8,9]. Consequently, clinical guidance for adolescents often relies on extrapolation from adult data or adopts conservative recommendations in the absence of adolescent-specific evidence [9-10].

Although creatine is widely used for performance enhancement in sports, existing literature primarily emphasizes performance-related outcomes and frequently extrapolates adult findings to younger populations, with limited focus on clinically relevant considerations such as safety monitoring, dosing strategies, and patient counseling [3,9,10]. Furthermore, inconsistent definitions of “athlete” and the underrepresentation of adolescents in clinical trials limit the applicability of current guidance to real-world clinical practice [9,10].

While several narrative reviews and expert position statements have addressed creatine supplementation in youth, few have systematically synthesized safety-related outcomes specifically within adolescent and physically active pediatric populations using contemporary evidence [9-11]. Prior reviews often combine pediatric, adolescent, and adult data or emphasize theoretical risks rather than empirically observed adverse events. A focused synthesis of renal, hepatic, and cardiometabolic safety outcomes in adolescent populations is therefore necessitated to better inform evidence-based clinical counseling, shared decision-making, and future research priorities.

Research question and study aims

The objective of this systematic review was to synthesize available evidence regarding the safety of creatine monohydrate supplementation in adolescent athletes and physically active adolescents.

## Review

Methods

Search Strategy

This systematic review was conducted in accordance with the Preferred Reporting Items for Systematic Reviews and Meta-Analyses (PRISMA) reporting guidelines. A formal review protocol was not prospectively registered (e.g., PROSPERO). A comprehensive literature search was performed in PubMed on December 24, 2025, using predefined eligibility criteria to identify relevant studies. Boolean operators were applied to refine the search strategy. The search string used was: (creatine monohydrate[Title/Abstract] OR creatine supplementation[Title/Abstract]) AND (adolescent OR adolescence OR youth OR pediatric) AND (safety OR adverse OR renal OR kidney OR toxicity).

Filters were applied to include only English-language articles published between January 1, 2015, and December 24, 2025. The search yielded 34 records. Duplicate records were removed prior to screening. Reference lists of included articles were manually reviewed to identify any additional relevant studies not captured in the initial search. The search strategy may not have captured all relevant studies due to limited use of Medical Subject Headings (MeSH) terms and synonyms related to safety outcomes, and because the search was restricted to a single database (PubMed), potentially introducing selection bias. A quantitative meta-analysis was not performed due to the small number and heterogeneity of included studies.

Inclusion Criteria

The study population included adolescents and physically active pediatric individuals, generally defined as ≤18 years of age, including both athletic and clinical populations. Studies with age ranges extending beyond 18 years were included only when adolescents constituted the majority of the study population and when results were not stratified by age. This approach was used to preserve relevant adolescent data but introduces potential selection bias and should be interpreted with caution. Eligible study designs included randomized controlled trials (RCTs), cohort studies, and longitudinal observational studies. The primary intervention of interest was creatine monohydrate supplementation, including fixed-dose, weight-based, or loading-maintenance regimens.

Studies were required to report safety-related outcomes, including renal function markers (e.g., serum creatinine), hepatic enzymes, hematologic parameters, cardiometabolic indices, or documented adverse events. Only articles published in English within the past 10 years (2015-2025) were considered for inclusion.

Exclusion Criteria

Studies were excluded if they focused exclusively on adult populations without separate adolescent subgroup data, evaluated creatine only as part of a multi-ingredient supplement without the ability to isolate creatine-specific safety outcomes, or reported solely on performance-related outcomes without safety data. Ineligible study designs included case reports, systematic reviews, narrative reviews, meta-analyses, editorials, conference abstracts without full text, and animal studies. Studies published in languages other than English or prior to 2015 were also excluded.

Study Selection and Screening

Rayyan software (Rayyan Systems Inc., Doha, Qatar) was used to conduct the screening process. Two independent reviewers screened titles and abstracts based on the predefined eligibility criteria. Full-text articles of potentially eligible studies were subsequently assessed independently by both reviewers.

Any disagreements regarding study inclusion were resolved through discussion and adjudication by a senior author. Studies were excluded for reasons including irrelevant population, absence of safety outcomes, ineligible study design, duplicate records, or inability to attribute findings specifically to creatine monohydrate supplementation. All exclusions were documented and reflected in the PRISMA flow diagram.

Data Extraction

Data extraction was performed independently by two reviewers using a standardized data collection form to ensure consistency. Extracted variables included author, year of publication, journal, study design, population characteristics (age range, sex, clinical or athletic status), sample size, creatine dosing protocol (dose, duration, loading or maintenance strategy), comparator group when applicable, duration of follow-up, safety-related laboratory and clinical outcomes, and reported adverse events.

Reporting Bias

A formal risk-of-bias or methodological quality assessment (e.g., Cochrane Risk of Bias Tool or Newcastle-Ottawa Scale) was not performed due to the limited number and heterogeneity of included studies. This represents a limitation and may affect the internal validity and interpretability of the findings.

Results

Study Selection and Characteristics

The literature search identified 34 records through PubMed database searching. No duplicate records were identified or removed prior to screening. All 34 records underwent title and abstract screening, of which 24 were excluded for not meeting inclusion criteria.

Nine full-text articles were sought for retrieval and successfully obtained. All nine reports were assessed for eligibility. Four studies were excluded at the full-text stage, including one due to unavailable full text and three due to inclusion of an ineligible population.

**Figure 1:**
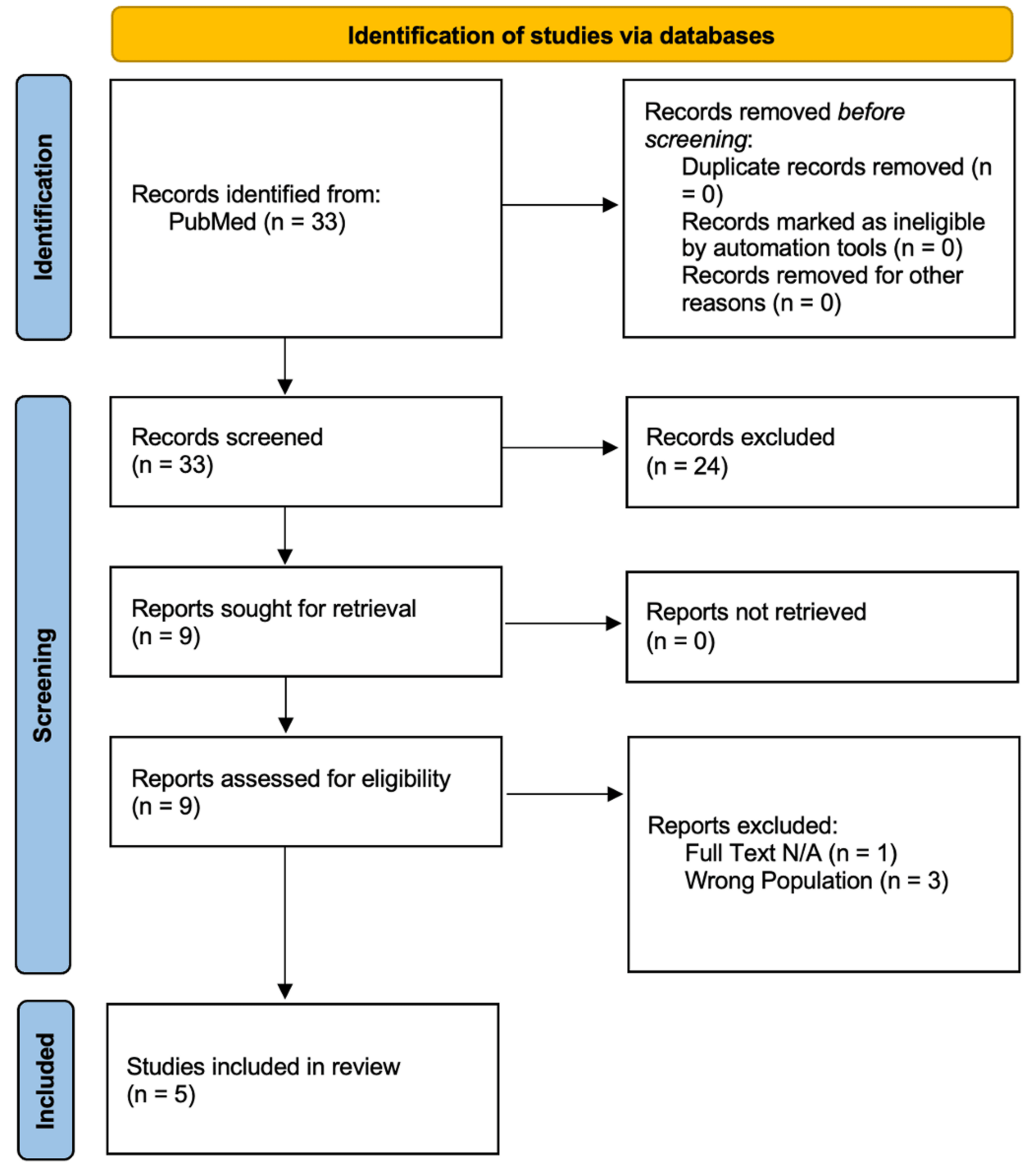
PRISMA Flow Diagram of Study Selection Process PRISMA 2020 flow diagram created using the PRISMA online tool, summarizing study selection and exclusion based on predefined criteria. PRISMA: Preferred Reporting Items for Systematic Reviews and Meta-Analyses

Ultimately, five studies met all predefined inclusion criteria and were included in the final qualitative synthesis (Figure 1). These comprised three randomized controlled trials and two observational cohort studies encompassing pediatric, adolescent, and young adult populations [12-16].

Study populations were heterogeneous and included youth athletes participating in organized football programs, adolescent females with selective serotonin reuptake inhibitor (SSRI)-resistant major depressive disorder, and patients with pediatric inflammatory myopathies, including juvenile dermatomyositis [12-16].

Creatine monohydrate dosing protocols varied across studies. Weight-based regimens included approximately 0.1 g/kg/day, while fixed daily dosing ranged from 2 to 10 g/day. Several trials employed loading-maintenance strategies consisting of short loading phases (e.g., 20 g/day) followed by maintenance dosing (e.g., 5 g/day) [12-14]. Interventional study durations ranged from six to 32 weeks [12-14,16]. The prospective cohort study assessed long-term cardiometabolic outcomes over approximately seven years following initiation of creatine-containing performance-enhancing supplements during adolescence [15].

Renal Safety

Renal safety was evaluated in three of the four included studies [12-14]. Across randomized controlled trials in pediatric and adolescent populations, no clinically meaningful changes were observed in serum creatinine, estimated glomerular filtration rate (eGFR), or other renal biomarkers [12-14]. In adolescents with SSRI-resistant depression, serum creatinine levels remained stable across creatine dosing groups compared with placebo over the eight-week intervention period [15].

Similarly, in an athletic cohort of youth and young adult female football players, renal biomarkers remained within reference ranges throughout the competitive season, and no creatine-related renal adverse events were reported [16].

Hepatic and Hematologic Safety

Hepatic safety outcomes were evaluated in the real-world athletic cohort study of youth and young adult female football players conducted by Garcia et al., in which liver enzyme concentrations, including alanine aminotransferase and aspartate aminotransferase, remained within established reference ranges throughout the competitive season [16]. No evidence of hepatotoxicity or clinically significant hematologic abnormalities attributable to creatine supplementation was reported during the study period. These findings suggest that creatine use in this athletic population was not associated with adverse hepatic safety outcomes within the observed timeframe.

Cardiometabolic Safety

Long-term cardiometabolic safety was evaluated in a nationally representative prospective cohort followed from adolescence into young adulthood [15]. After adjustment for demographic characteristics, health behaviors, and baseline cardiometabolic risk factors, no significant associations were identified between legal performance-enhancing substance use, including creatine-containing supplements, and adverse cardiometabolic outcomes [15]. These outcomes included body mass index, blood pressure, glycemic markers, lipid profiles, and the incidence of diabetes, hypertension, or hyperlipidemia [15].

Adverse Events and Tolerability

Across all included studies, creatine monohydrate supplementation was described as well-tolerated, with no serious adverse events attributed to supplementation [12-15]. Randomized trials in pediatric and adolescent populations reported no significant differences in adverse events or laboratory abnormalities between creatine and placebo groups [12-14].

In the athletic cohort, elevations in creatine phosphokinase were observed; however, these findings were attributed to training load rather than supplementation. Adherence rates were high across clinical trials, supporting the feasibility and short-term tolerability of creatine supplementation in pediatric and adolescent populations [12-14].

Overall, across the five included studies, creatine monohydrate supplementation in pediatric, adolescent, and young adult populations was not associated with clinically meaningful renal, hepatic, hematologic, or cardiometabolic safety concerns during the reported follow-up periods [12-16]. Long-term observational data similarly did not demonstrate increased cardiometabolic risk associated with creatine-containing supplement use initiated during adolescence [15,16].

Discussion

This systematic review synthesized contemporary evidence evaluating the safety of creatine monohydrate supplementation in adolescent and physically active pediatric populations. Across four eligible studies representing randomized controlled trials, real-world athletic cohorts, and long-term observational data, creatine supplementation was not associated with consistent short-term adverse safety signals in renal, hepatic, hematologic, or cardiometabolic domains (Table 1).

**Table 1:** Summary of Included Studies Evaluating the Safety and Clinical Outcomes of Creatine Supplementation in Pediatric and Adolescent Populations SSRI: selective serotonin reuptake inhibitor; PES: performance-enhancing substances; BMI: body mass index; HbA1c: hemoglobin A1c; GFR: glomerular filtration rate; ALT: alanine aminotransferase; AST: aspartate aminotransferase; CPK: creatine phosphokinase; 31P-MRS: phosphorus-31 magnetic resonance spectroscopy.

Title	Reference	Study Design	Population	Age Group	Creatine Dose	Duration	Safety Outcomes Assessed	Key Safety Findings
Creatine target engagement with brain bioenergetics: a dose-ranging phosphorus-31 magnetic resonance spectroscopy study of adolescent females with SSRI-resistant depression	Kondo DG, Forrest LN, Shi X, et al. (2016) [12]	Randomized, placebo-controlled, dose-ranging trial	Adolescent females with SSRI-resistant major depressive disorder	Adolescents	Creatine monohydrate 2 g/day, 4 g/day, or 10 g/day	8 weeks	Adverse events, weight change, serum creatinine; neuroimaging biomarkers (31P-MRS phosphocreatine)	No significant differences in adverse events, weight gain, or serum creatinine between creatine and placebo groups. Creatine increased cerebral phosphocreatine in a dose-dependent manner without evidence of renal or metabolic safety concerns.
Efficacy and safety of creatine supplementation in juvenile dermatomyositis: A randomized, double-blind, placebo-controlled crossover trial.	Solis MY, Hayashi AP, Artioli GG, et al. (2015) [13]	Randomized, double-blind, placebo-controlled crossover trial	Patients with juvenile dermatomyositis	Pediatric / adolescents	Creatine monohydrate 0.1 g/kg/day	12 weeks	Renal function tests, laboratory safety parameters, reported adverse effects	Creatine supplementation was well tolerated. No adverse effects were reported, and kidney function remained unaffected throughout the study period.
The Effect of Creatine Supplementation on Muscle Function in Childhood Myositis: A Randomized, Double-blind, Placebo-controlled Feasibility Study.	Dover S, Stephens S, Schneiderman JE, et al. (2020) [14]	Randomized, double-blind, placebo-controlled feasibility trial	Children with juvenile dermatomyositis	Pediatric / adolescents	Creatine supplementation (dose per protocol)	6 months	Adverse effects, tolerability, adherence, laboratory safety parameters	Creatine supplementation was safe and well tolerated. No significant adverse effects were observed during the 6-month study period, and adherence was high.
Associations between legal performance-enhancing substance use and future cardiovascular disease risk factors in young adults: A prospective cohort study. PLoS One.	Nagata JM, Ganson KT, Cunningham ML, et al. (2020) [15]	Prospective longitudinal cohort study (Add Health)	Nationally representative adolescents followed into young adulthood	Adolescents / young adults	Self-reported legal PES use (including creatine monohydrate)	7-year follow-up	Cardiometabolic outcomes (BMI, blood pressure, HbA1c, lipid profile, diabetes, hypertension, hyperlipidemia)	No significant prospective associations between legal PES use and cardiometabolic risk factors over 7 years. Creatine-containing PES use was not associated with adverse cardiometabolic outcomes after adjustment for confounders.
Safety of long-term creatine supplementation in women's football players: a real-world in-season study	Garcia MP, Longobardi I, Saito T, et al. (2025) [16]	Real-world longitudinal single-arm study	Female football (soccer) players from youth and professional teams (n=71)	Adolescents / young adults	Creatine monohydrate loading phase 20 g/day for 7 days, followed by 5 g/day maintenance	32 weeks (in-season)	Hematological markers; renal function (creatinine, urea, GFR, albuminuria); hepatic function (ALT, AST); biochemical safety markers	No clinically meaningful adverse effects observed. Minor fluctuations in some biochemical markers occurred but remained within normal reference ranges. Renal and hepatic function were unaffected. Elevated CPK likely reflected training load rather than creatine supplementation.

Renal safety remains one of the most frequently cited concerns regarding creatine use in adolescents. Across randomized and cohort studies included in this review, no clinically meaningful changes were observed in serum creatinine, eGFR, or other renal biomarkers. Elevations in serum creatinine, when present in athletic populations, must be interpreted cautiously because creatine supplementation can increase creatinine production through nonpathologic conversion without reflecting intrinsic renal dysfunction. The absence of sustained renal impairment across short- and medium-term trials provides reassurance within the studied dosing ranges and durations.

Hepatic safety data, though more limited, similarly demonstrated no evidence of hepatotoxicity. Liver enzyme concentrations remained within reference ranges in the real-world athletic cohort, supporting the short-term hepatic safety of creatine supplementation during periods of high training load. Hematologic fluctuations reported in select trials were minor and not clinically significant.

Long-term cardiometabolic safety data, derived from a nationally representative cohort followed for approximately seven years, did not demonstrate associations between adolescent creatine-containing supplement use and adverse outcomes such as hypertension, dyslipidemia, diabetes, or elevated body mass index. While observational in nature and not exclusive to creatine monotherapy, these findings provide important real-world context regarding longer-term health trajectories.

The included study populations were heterogeneous and encompassed both clinical and athletic cohorts, including adolescents with psychiatric and inflammatory conditions. This heterogeneity may limit the generalizability of findings to healthy adolescent athletes, as baseline health status and concurrent therapies may influence safety outcomes.

An important real-world consideration not captured in most included studies is variability in over-the-counter supplement quality. Commercial creatine products may differ in purity, dosing accuracy, and potential contamination with other substances. These factors may influence safety outcomes in real-world settings and limit direct extrapolation of findings from controlled research environments.

The long-term cardiometabolic findings were derived from a large observational cohort assessing self-reported use of legal performance-enhancing substances, including but not limited to creatine. As exposure was not specific to creatine monotherapy and relied on self-reported data, causal inference regarding creatine alone is limited.

Limitations and Future Directions

Several limitations should be acknowledged. The number of eligible studies was small, and most interventional trials were short in duration, limiting conclusions regarding long-term safety. Study populations were heterogeneous and included both clinical and athletic cohorts, which may reduce generalizability to healthy adolescent athletes. Additionally, the inclusion of studies with mixed-age populations and reliance on non-creatine-specific observational data introduces potential bias.

The search strategy was limited to a single database (PubMed), which may have resulted in omission of relevant studies indexed in other databases such as Embase, Scopus, or the Cochrane Library. Furthermore, a formal risk-of-bias or methodological quality assessment was not performed, which limits the ability to evaluate internal study validity. Variability in over-the-counter supplement quality, including potential contamination and inconsistent dosing, was not assessed in the included studies and represents an additional real-world limitation.

Future research should prioritize larger, adequately powered randomized trials in healthy adolescent athletes, longer-term follow-up periods, standardized dosing protocols, and systematic monitoring of laboratory and clinical safety outcomes.

## Conclusions

This systematic review summarizes currently available evidence evaluating the safety of creatine monohydrate supplementation in adolescent and physically active pediatric populations. Across the included studies, no consistent short-term safety signals were identified in renal, hepatic, hematologic, or cardiometabolic outcomes within the studied durations. However, these findings should be interpreted cautiously given the small number of studies, heterogeneity of included populations, limited long-term data, and methodological constraints. Further well-designed prospective studies with standardized dosing protocols and extended follow-up durations are needed to more definitively characterize the safety profile of creatine supplementation in adolescent populations.
